# Initial experience at a university teaching hospital from using telemedicine to promote education through video conferencing

**DOI:** 10.1590/S1516-31802012000100006

**Published:** 2012-02-13

**Authors:** Bruno Monteiro Tavares Pereira, Thiago Rodrigues Araújo Calderan, Marcos Tadeu Nolasco da Silva, Antonio Carlos da Silva, Antonio Carlos Marttos, Gustavo Pereira Fraga

**Affiliations:** I MD. Attending Physician, Division of Trauma Surgery, Faculdade de Ciências Médicas, Universidade Estadual Campinas (FCM - Unicamp), Campinas, São Paulo, Brazil. Universidade Estadual de Campinas Division of Trauma Surgery Faculdade de Ciências Médicas Universidade Estadual Campinas Campinas São Paulo Brazil; II MD. PhD. Assistant Professor, Telemedicine Program Coordinator, Faculdade de Ciências Médicas, Universidade Estadual Campinas (FCM - Unicamp), Campinas, São Paulo, Brazil. Universidade Estadual de Campinas Faculdade de Ciências Médicas Universidade Estadual Campinas Campinas São Paulo Brazil; III Technician in Computing and Telemedicine, Universidade Estadual Campinas (FCM - Unicamp), Campinas, São Paulo, Brazil. Universidade Estadual de Campinas Universidade Estadual Campinas Campinas São Paulo Brazil; IV MD. Assistant Professor, Division of Trauma Surgery and Surgical Critical Care; and Head of Telemedicine, Ryder Trauma Center, Jackson Memorial Hospital, University of Miami, Miami, Florida, United States. University of Miami Division of Trauma Surgery and Surgical Critical Care University of Miami Miami Florida USA; V MD, PhD. Professor of Surgery and Head of the Division of Trauma Surgery, Faculdade de Ciências Médicas, Universidade Estadual Campinas (FCM - Unicamp), Campinas, São Paulo, Brazil. Universidade Estadual de Campinas Division of Trauma Surgery Faculdade de Ciências Médicas Universidade Estadual Campinas Brazil

**Keywords:** Telemedicine, Education, distance, Technology, Telecommunications, Videoconferencing, Telemedicina, Educação à distância, Technologia, Telecomunicações, Videoconferência

## Abstract

**CONTEXT AND OBJECTIVE:**

Telehealth and telemedicine services are advancing rapidly, with an increas ing spectrum of information and communication technologies that can be applied broadly to the popula tion’s health, and to medical education. The aim here was to report our institution’s experience from 100 videoconferencing meetings between five different countries in the Americas over a one-year period.

**DESIGN AND SETTING:**

Retrospective study at Universidade Estadual de Campinas.

**METHODS:**

Through a Microsoft Excel database, all conferences in all specialties held at our institution from September 2009 to August 2010 were analyzed retrospectively. RESULTS: A total of 647 students, physicians and professors participated in telemedicine meetings. A month ly mean of 8.3 (± 4.3) teleconferences were held over the analysis period. Excluding holidays and the month of inaugurating the telemedicine theatre, our teleconference rate reached a mean of 10.3 (± 2.7), or two teleconferences a week, on average. Trauma surgery and meetings on patient safety were by far the most common subjects discussed in our teleconference meetings, accounting for 22% and 21% of the total calls.

**CONCLUSION:**

Our experience with telemedicine meetings has increased students’ interest; helped our institution to follow and discuss protocols that are already accepted worldwide; and stimulated professors to promote telemedicine-related research in their own specialties and keep up-to-date. These high-tech nology meetings have shortened distances in our vast country, and to other reference centers abroad. This virtual proximity has enabled discussion of international training with students and residents, to increase their overall knowledge and improve their education within this institution.

## RESUMO

**CONTEXTO E OBJETIVO:**

Serviços de telessaúde e telemedicina estão avançando rapidamente, com um espectro cada vez maior de tecnologias da informação e comunicação que podem ser aplicadas de forma ampla para a saúde da população, bem como para a educação médica. O objetivo deste artigo é relatar a experiência da nossa instituição com 100 reuniões por videoconferência entre cinco diferentes países das Américas no período de um ano.

**TIPO DE ESTUDO E LOCAL:**

Estudo retrospectivo na Universidade Estadual de Campinas.

**MÉTODOS:**

Através de um banco de dados do Microsoft Excel, foram analisadas retrospectivamente to das as conferências realizadas em nossa instituição, de setembro de 2009 a agosto de 2010, em todas as especialidades.

**RESULTADOS:**

Um total de 647 alunos, médicos e professores participaram das reuniões de telemedicina. Em média, 8,3 (± 4,3) teleconferências foram realizadas mensalmente durante o período analisado. Excluin do os feriados e o mês de inauguração do anfiteatro de telemedicina, as nossas taxas de teleconferência atingiram a média de 10,3 (± 2,7), ou duas teleconferências sobre uma média semanal. Cirurgia do Trauma e reuniões sobre segurança dos pacientes foram, de longe, os temas mais comuns discutidos em nossas reuniões de teleconferência, correspondendo por 22% e 21% do total de chamadas.

**CONCLUSÃO:**

Nossa experiência com as reuniões de telemedicina aumentou o interesse dos alunos, ajudou a nossa instituição a acompanhar e discutir protocolos que já são aceitos em todo o mundo e estimulou nossos professores a promover pesquisas relacionadas à telemedicina em suas próprias espe cialidades, mantendo-os atualizados. Essas reuniões com envolvimento de alta tecnologia encurtaram as distâncias dentro de nosso país vasto e com outros centros de referência no exterior. Esta proximidade virtual permitiu discussões com alunos e residentes sobre estágios internacionais a fim de aumentar seu conhecimento global e melhorar a sua educação dentro da própria instituição.

## INTRODUCTION

Around the year 300 B.C., when Hippocrates of Kos separated the discipline of medicine from religion, believing and arguing that disease was not a punishment inflicted by the gods, but rather, the product of environmental factors, diet and living habits, the brightest philosopher living at that time would perhaps never have foreseen how technology would play such an important role in medicine nowadays. Many different forms of technologies have been applied to and used by medicine since that time, and telemedicine is one of the most recent ones. Telemedicine today is vastly different from its humble origins in the Netherlands in the early 1900s, as well as medicine and medical care in general.^1^

Telehealth science and technology services are advancing rapidly, with an increasing spectrum of information and communication technologies that can be applied broadly to the population’s health, as well as to care for individual patients.^2^ Telemedicine and telehealth science have already been used in the fields of military medicine, disaster management, emergency preparedness, telepresence technologies and robotic medicine, in geographically remote zones and in many different medical fields and areas.^3^^,^^4^^,^^5^^,^^6^^,^^7^^,^^8^^,^^9^^,^^10^^,^^11^

Telemedicine may be defined as the use of telecommunications and information technology to support the delivery of healthcare at a distance.^12^ However, telemedicine has also appeared to be very useful for delivering distance education through many ways, including videoconferencing.^13^ Through the use of telemedicine technology, videoconferencing combines audio and video to provide a means for efficient communication, collaboration and decision-making in real time. It enables people who cannot be physically present in the same location to conduct a face-to-face meeting. Videoconferencing enables each participant to be able to see each other’s facial expressions and body language. It also allows people to share files and data, so that it is easy to hold presentations, review documents and make fast decisions. This so called telemedicine technology is very easy to implement, even in developing countries. In Brazil, for instance, the government has joined with the Ministry of Science and Technology and invested in the creation of a telemedicine network named RUTE (University Network of Telemedicine, available from http://rute.rnp.br). Its aim is to connect several university hospitals in the country, including all specialties, into a single information network.

## OBJECTIVE

The objective of this study was to report our institution’s experience from 100 videoconferencing meetings between five different countries in the Americas over a one-year period.

## METHODS

The Clinical Hospital of Universidade Estadual de Campinas (Unicamp) is a teaching facility located in Campinas, a city in the State of São Paulo, Brazil. This is a 500-bed tertiary-level institution, with all medical specialties and surgical services represented. The area covered by this facility includes the entire Metropolitan Region of Campinas, with approximately four million inhabitants.

Through a prospective framed Microsoft Excel database, compiled by our information technology (IT) team, we retrospectively analyzed all of the conferences with five different countries that were held in our institution from September 2009 to August 2010, within all different specialties. The main objective of the videoconferences was to promote education through exchanging concepts, ideas and care management in different specialties, among five different countries in the Americas (United States, Colombia, Chile, Puerto Rico and Brazil) and/or among multiple sites in different states or municipalities in two of these countries (Brazil and the United States, or Brazil only). Residents and staff were previously advised to prepare a real case presentation lecture on Microsoft PowerPoint software or Macintosh Keynote software to be discussed by the videoconference participants.

**Figures 1and 2.**
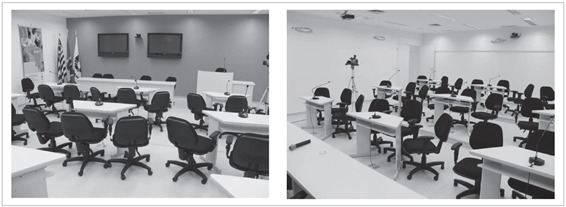
Teleconference theatre at Universidade Estadual de Campinas (Unicamp).

The conferences were carried out using the same videoconference equipment/technology (Tandberg 6000 megapixels, New York, United States), supervised by the same IT technician. Our institution’s teleconference theatre is able to hold 36 seated attendees, who may be teaching staff or students. It is also relatively new, with less than two years of use, and the equipment available includes a video camera suitable for videoconferencing (Tandberg HD, New York, United States), a codec (Tandberg HD MXP 6000, New York, United States), two LCD monitors and surround sound (Figures 1 and 2).

We excluded all testing conference calls.

## RESULTS

**Figure 3.**
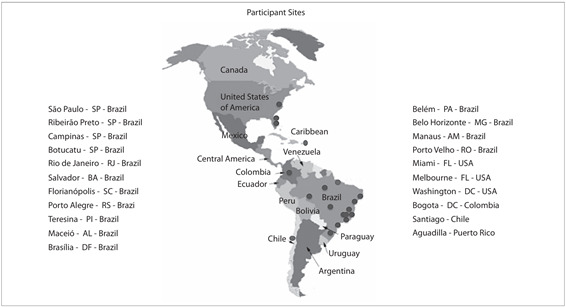
Telemedicine videoconferencing sites involved.

During the one-year period, the Clinical Hospital of Unicamp held 100 conferences with multiple national sites (Brazil) and/or one to four other countries abroad. Figure 3 shows all the conference sites involved. A total of 647 registered participants were exposed to telemedicine education conferences. Most of them were healthcare personnel with confirmed presence in more than one meeting. All the participants declared that the telemedicine conferences had, in some manner, increased their knowledge.

**Figure 4.**
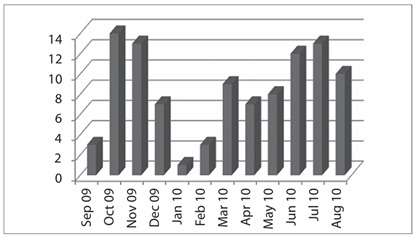
Number of videoconferences held, according to month.

**Figure 5.**
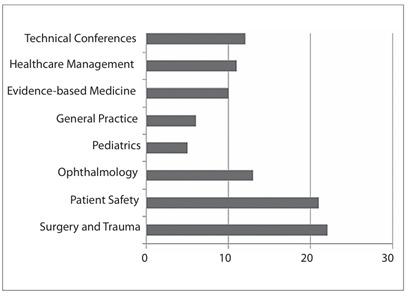
Healthcare fields involved, according to number of meetings.

The healthcare fields involved in the videoconferences and their frequencies can be seen in Figures 4 and 5, respectively. A mean of 8.3 (± 4.3) teleconferences was held every month over the analysis period. Excluding holiday months (January and February 2010) and the month of inaugurating the telemedicine theatre (September 2009), our teleconference rates reached a mean of 10.3 (± 2.7), or two teleconferences a week, on average. Trauma surgery and meetings on patient safety were by far the most common subjects discussed in our teleconferences, accounting for 22% and 21% of all of the calls. The Division of Trauma Surgery had the largest number of international conferences. A mean of 5.4 (± 1.4) connected sites were present in tele-trauma videoconferences.

Telemedicine is now part of the trauma surgery residents’ curriculum and medical students’ surgery rotation in our institution. Weekly meetings are happening regularly (every Friday) together with at least one international site (Ryder Trauma Center, Jackson Memorial Hospital, University of Miami).

## DISCUSSION

Telemedicine has already proven to be effective.^14^^,^^15^ Rapidly advancing technology is forcing researchers to look for new, different telemedicine and telehealth applications, thereby enlarging the telemedicine spectrum to all fields of medicine, as well as having concern for ethics and safety.^16^ The future looks promising. Every day, more options for telemedicine and telehealth appear in the worldwide media, in order to do away with outpatient follow-ups.^17^^,^^18^^,^^19^^,^^20^^,^^21^ Speculation regarding performing surgery in space even appears to be coming closer to reality.^22^ We feel that our experience with telemedicine videoconferencing has increased students’ interest and has helped our institution to follow and discuss protocols that are already accepted worldwide. It have stimulated our teaching staff to promote telemedicine-related research within their own specialties and keep up-to-date. These high-tech videoconferences have shortened distances within our country of 8,459,417 square kilometers and have brought other reference centers abroad closer to us. This virtual proximity has allowed students and residents at our institution to participate in observership/fellowship programs in order to increase their overall knowledge and improve their on-site education. Furthermore, in a big country like Brazil, where the distances are overwhelming and there are still some remote places that can only be reached by boat, telemedicine plays an important role in delivering healthcare, healthcare providers’ support and medical education.^23^

With regard to the number of conference calls, trauma surgery was the leading discipline in the telemedicine videoconferences. It is not an overstatement to say that, although medicine is not an exact science and thus different approaches are well tolerated, all the services interlinked within this group can now “speak the same language” when talking about their specialties. Weekly tele-trauma meetings are being conducted regularly with multiple national and international sites. Having a close connection to international trauma reference centers is clearly a stimulus for improving our patients’ quality of care. Over the past years, trauma telemedicine has evolved and it is now becoming integrated into trauma and surgical care.^12^^,^^24^ Latifi et al.^24^ retrospectively evaluated 59 trauma and general surgery patients at rural hospitals, by means of telemedicine, from their level I trauma center. The telepresence of a trauma surgeon was considered potentially lifesaving for six patients (10.2%) and the authors concluded that telemedicine improved outcomes and reduced trauma care costs.^24^

## CONCLUSION

As a final point, we strongly believe that telemedicine has come to stay. It has played an important role in our institution, through enriching the education and pushing our students and teaching staff to a higher standard at the international level. In the future, we expect to enroll more disciplines into telemedicine videoconferencing; create new groups for telemedicine discussions; make more use of telemedicine technologies that deliver telehealth care and tele-education into the remotest areas; and integrate telemedicine into the medical school curriculum, in the same way that some other reference centers have already done.^25^
